# Hydroxymethyluracil modifications enhance the flexibility and hydrophilicity of double-stranded DNA

**DOI:** 10.1093/nar/gkv1199

**Published:** 2015-11-17

**Authors:** Spencer Carson, James Wilson, Aleksei Aksimentiev, Peter R. Weigele, Meni Wanunu

**Affiliations:** 1 Department of Physics, Northeastern University, Boston, MA, USA; 2 Department of Physics, University of Illinois at Urbana-Champaign, Urbana, IL, USA; 3 New England Biolabs, Ipswich, MA, USA; 4 Department of Chemistry and Chemical Biology, Northeastern University, Boston, MA, USA

## Abstract

Oxidation of a DNA thymine to 5-hydroxymethyluracil is one of several recently discovered epigenetic modifications. Here, we report the results of nanopore translocation experiments and molecular dynamics simulations that provide insight into the impact of this modification on the structure and dynamics of DNA. When transported through ultrathin solid-state nanopores, short DNA fragments containing thymine modifications were found to exhibit distinct, reproducible features in their transport characteristics that differentiate them from unmodified molecules. Molecular dynamics simulations suggest that 5-hydroxymethyluracil alters the flexibility and hydrophilicity of the DNA molecules, which may account for the differences observed in our nanopore translocation experiments. The altered physico-chemical properties of DNA produced by the thymine modifications may have implications for recognition and processing of such modifications by regulatory DNA-binding proteins.

## INTRODUCTION

Epigenetic modifications in eukaryotic DNA, which are most commonly manifested in cytosine (C) taking the form of 5-methylcytosine (mC) and its oxidized product 5-hydroxymethylcytosine (hmC), have been implicated in gene expression, genetic imprinting and other various genetic processes (1,2). Less abundant than cytosine modifications, several oxidized products of thymine (T) including 5-hydroxymethyluracil (hmU), formed by ionizing radiation (3), reactive oxygen species (4) or Tet enzymes (5) have been correlated with chronic inflammatory diseases and cancers (6). Although these thymine lesions can be detected in bulk solution using mass spectroscopy (7,8), little is known about their impact on DNA structure. Nanopores have emerged as single-molecule probes for high-resolution determination of base composition in double-stranded DNA (9–11)(dsDNA) and single-stranded DNA (12–14) (ssDNA), which have made nanopores a prime candidate for next-generation DNA mapping and sequencing applications (15).

**Figure 1.**
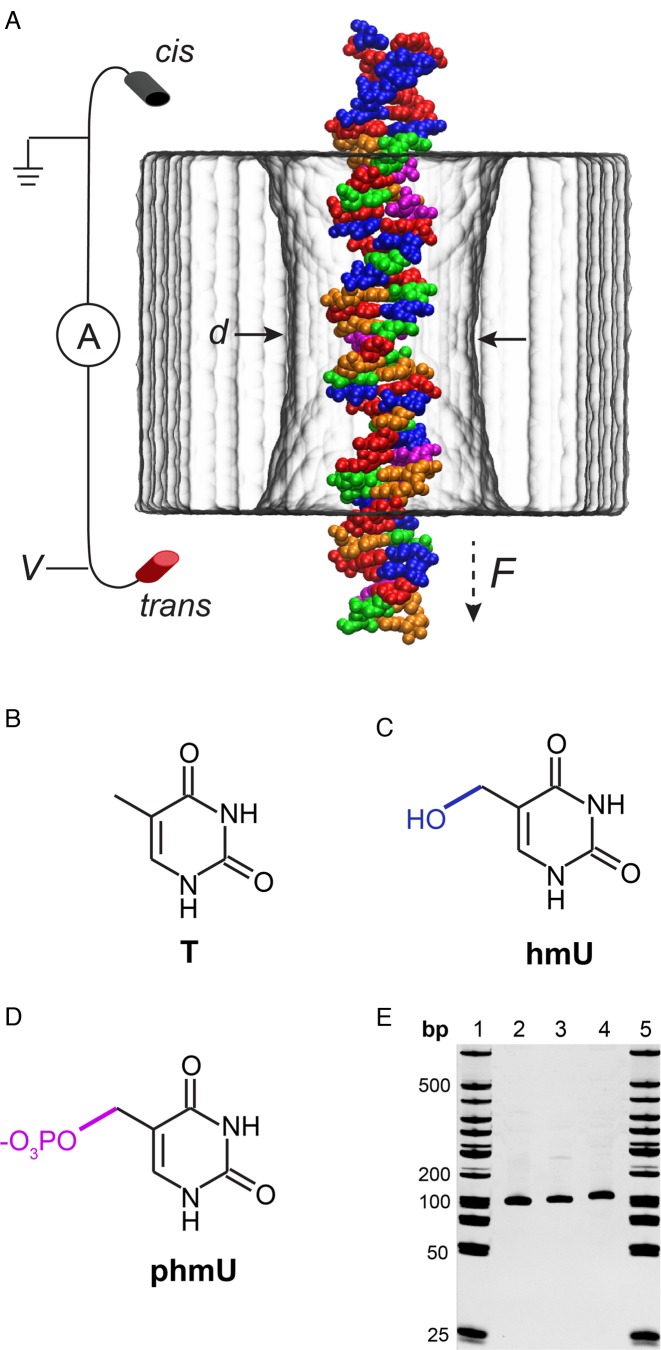
Nanopore detection of thymine variants in DNA. (**A**) A microscopic model of a DNA translocation experiment featuring a 3.5 nm diameter SiN nanopore (gray) and a fragment of p-DNA. The DNA molecule is colored according to its nucleotide content: red corresponds to adenine nucleotides (A), green to cytosines (C), orange to guanines (G), blue to 5-hydroxymethyluracil (hmU) and magenta to phosphorylated 5-hydroxymethyluracil (phmU). (**B**–**D**) Chemical structures of thymine (T) and its hmU and phmU variants. The modifications are highlighted in blue and magenta. (**E**) Gel electrophoresis of 101-bp DNA samples (20% PAGE, lanes 1 and 5: 50 bp dsDNA ladder; 2: t-DNA; 3: h-DNA; 4: p-DNA).

A nanometer-size aperture in a thin membrane—a nanopore—provides a solvent-filled passage between two solution compartments separated by the membrane. When subjected to a trans-membrane voltage, individual molecules of DNA can transit from a negatively biased compartment into a positively biased one through the nanopore, as depicted in Figure 1A. The presence of DNA in a nanopore is experimentally detected as a transient drop in ionic current flowing through the nanopore. Each translocation event is characterized by the duration of the ionic current blockade, or dwell time (*t_d_*), and the amount of ion current reduction, or current blockade (*ΔI*). The mean values of *t_d_* and *ΔI* determined from a population of single-molecule transport events (typically at least 1000) reflect properties of the molecules, e.g. their contour length (16–19), cross-sectional diameter (20,21) and microscopic conformation (17,22).

Recently, various approaches to differentiating dsDNA fragments that contain cytosine and modified cytosines have been pursued using small diameter (≈4 nm) silicon nitride (SiN) nanopores (10,23). In these studies, DNA flexibility accounted for differences in transport dynamics, and has been previously investigated using experimental and computational tools (24,25). In addition, nanopore identification of single cytosine modifications was demonstrated using methylation-specific binding proteins (26), streptavidin-based ssDNA immobilization (27), and DNA polymerases acting as molecular ratchets (28,29). In this article, we characterize the transport kinetics of the following DNA variants: (I) canonical DNA containing no thymine modifications (t-DNA), (II) DNA where all thymine nucleotides were replaced by hmU (h-DNA), and (III) h-DNA molecules that were further modified to have all TG sites phosphorylated (p-DNA) (see Materials and Methods for sample preparation information and Supplementary Figure S1 for DNA sequences). We show that thymine conversion to hmU and phosphorylated hmU (phmU) induces noticeable structural differences that affect transport of dsDNA through ultrathin SiN pores (14,21). Molecular dynamics (MD) simulations characterize the effect of the modifications on the equilibrium properties of DNA and relate the effect of modifications to the outcome of nanopore measurements.

## MATERIALS AND METHODS

### Nanopore fabrication and uncertainty determination

The fabrication of solid-state nanopore devices follows the procedure described in our previous publication (19). The specified uncertainties of *ΔI/I_o_* represent the standard deviations determined from Gaussian fits, and the uncertainties of *t_d_* represent the standard errors of the mean. The uncertainties reported for *v* and *D* are calculated using a standard bootstrapping procedure, as explained in earlier work (19).

### DNA sample preparation

The 101-bp sequence used for these studies is displayed in Supplementary Figure S1. Within this sequence there are 57 thymine sites that are enzymatically modified to hmU in h-DNA and 10 sites that are additionally modified to phmU (with 47 sites remaining hmU) in p-DNA. The DNA fragments t-DNA and h-DNA were prepared by polymerase chain reaction amplification using dNTP mixtures composed of either canonical nucleotides or a mixture with deoxy-hmUTP fully replacing dTTP. Fragments containing phmU (p-DNA) were prepared by treating the h-DNA with a kinase that specifically phosphorylates the hydroxymethyl moiety of an hmU 5′ to a guanine. PhmU is ordinarily formed as an intermediate to base hypermodification during the morphogenesis of the bacteriophages SP10 and ΦW14 (1). The kinase catalyzing the formation of phmU from ATP and hmUpG in polymeric DNA has recently been purified and shown to function *in vitro*. (P. Weigele, manuscript in preparation)

### Molecular dynamics simulations

All MD simulations were performed using the NAMD2 (30) software package using the CHARMM36 (31) force field for nucleic acids, water and ions. Ionic interactions included NBFIX corrections (32); a custom force field was used to describe the Si_3_N_4_ (33). The Si_3_N_4_ parameters were derived from first principle parameterization (34) and modified to be compatible with the CHARMM force field. The parameters for the hmU and phmU modifications were obtained using the CHARMM general force field library (35) and are included in the Supplementary Data.

Periodic boundary conditions were used along with the particle mesh Ewald method for long-range interactions electrostatics (36). Local interactions were calculated every time step, and the full electrostatic calculation was performed every three time steps using a multiple time-stepping scheme (37). A 2 fs time step was used with RATTLE (38) and SETTLE (39) algorithms applied to covalent bonds involving hydrogen atoms in DNA and water, respectively. The van der Waals forces were smoothly cut off starting at 10 Å and were cut off completely at 12 Å. A Nose-Hoover Langevin piston (40) was used for pressure control in NPT (i.e. constant number of particles, pressure and temperature) simulations with a period of 400 fs and a damping time scale of 200 fs. The temperature was controlled by a Langevin thermostat acting on the membrane atoms with a damping constant of 1.0 ps^−1^. In our free solution simulations, the Langevin thermostat acted on water oxygen atoms with a damping constant of 0.5 ps^−1^. To induce a transmembrane potential, a constant electric field was applied such that *E = –ΔV/l_z_*, where *ΔV* was the target transmembrane bias and *l_z_* was the length of the simulation system normal to the membrane (the *z*-axis in our system) (33,41). In accordance with a previously established protocol (42), each atom at the surface or in the interior of the membrane was harmonically restrained with a spring constant of 695 or 69.5 pN/Å, respectively, to give the membrane material a relative bulk permittivity of 7.5.

The atomic-scale model of the silicon nitride nanopore was built as in our previous work (19). The nanopore was made by removing atoms from a 7 nm thick crystalline silicon nitride membrane, so that the final nanopore had an hourglass shape with a minimum diameter of 3.5 nm. The DNA fragments were created using the 3D-DART web server (43) in a BDNA conformation. The 36-bp DNA fragments were then placed coaxial with the pore axis. Water was added to the open nanopore and DNA/nanopore systems with the solvate plug-in for VMD, and then ions were added to a target KCl concentration of 0.4 M using the autoionize plug-in for VMD. Each DNA/nanopore system was then equilibrated under NPT for 1 ns using a Langevin piston. Following equilibration, the system was simulated in the NVT ensemble (i.e. constant number of particles, volume and temperature) at a 200 mV transmembrane bias for 55 ns, which was sufficient for the number of ions to reach a constant value in the nanopore. The DNA sequence was chosen to reproduce a 36-bp fragment of the DNA used in the experiment or its variants (listed below).

t-DNA: CCATTCTTCCAAGTAGCTGAGTCTATGTGGATTTTA
h-DNA: CCAHHCHHCCAAGHAGCHGAGHCHAHGHGGAHHHHA
p-DNA: CCAHHCHHCCAAGHAGCPGAGHCHAHGPGGAHHHHA

Each DNA/nanopore system was then copied to make five independent simulations, each subject to an additional 2 ns equilibration in the NPT ensemble. The fifteen systems were simulated for 140 ns each under a 200 mV transmembrane bias in the NVT ensemble. The first 20 ns of each trajectory were discarded to ensure the ions were well equilibrated; the final 120 ns of each simulation were used to measure the blockade current. Each backbone phosphorous atom of the DNA was harmonically restrained to maintain a radial distance of 9.45 Å from the nanopore axis, and to not move along the nanopore. The spring constant of each restraint was chosen to be 6.95 pN/Å based on several short simulations. This choice of restraints allowed the DNA to alter its conformation as much as possible while remaining in the center of the nanopore. The application of such restraints maintained the DNA conformation coaxial with the nanopore while allowing the DNA to rotate about its axis.

The simulations of DNA in free solution were performed starting from the DNA conformations obtained at the end of the nanopore simulations. The molecules were placed into a cubic box, 13.7 nm on each side, and solvated with a 0.4 M KCl solution. Three free solution equilibration trajectories of ≈120 ns each were obtained for each of the three DNA variants (nine simulations in total). The simulations were run at a constant pressure of 1 atm and temperature of 295 K as described above. The trajectories were then analyzed using the 3DNA program (36), discarding the first 10 ns of each trajectory.

In two of the nine trajectories, a localized break in the DNA structure developed near the end of the simulation. As we were predominantly interested in characterization of near-equilibrium fluctuations of the DNA conformation, the part of the trajectory following the breaks was excluded from the analysis (see further details in the Supplementary Data). The mean value and the standard deviation of the intra- and inter-base pair parameters were calculated for each base pair (or pair of base pairs). The last 2–3 base pairs at each end of the molecule frayed during the simulations, making the bases intermittently unpaired. As such unpaired bases are not suitable for 3DNA analysis, five terminal base pairs at each end of each molecule were excluded from the analysis. The individual base pair values were averaged over the entire construct (excluding the five terminal base pairs). Doing so was justified by the high density of the modifications, which were present in at least every third base pair.

## RESULTS AND DISCUSSION

Figure 1 schematically illustrates the process of DNA translocation through a nanopore (Figure 1A), the chemical structures of the modified bases (Figure 1B–D) and gel electrophoresis of the products (Figure 1E). A low voltage bias (i.e. 200 mV) applied across a nanopore generates a steady-state ion current. Addition of DNA to the *cis* (-) chamber leads to a stochastic set of downward current spikes caused by individual DNA molecules transporting across the pore. Detection and analysis of each event using Pythion (www.github.com/rhenley/Pyth-Ion) yields the dwell time *t_d_* and the fractional current blockade *ΔI/I_o_*, which reflect DNA properties such as diameter, length, base content and flexibility.

**Figure 2.**
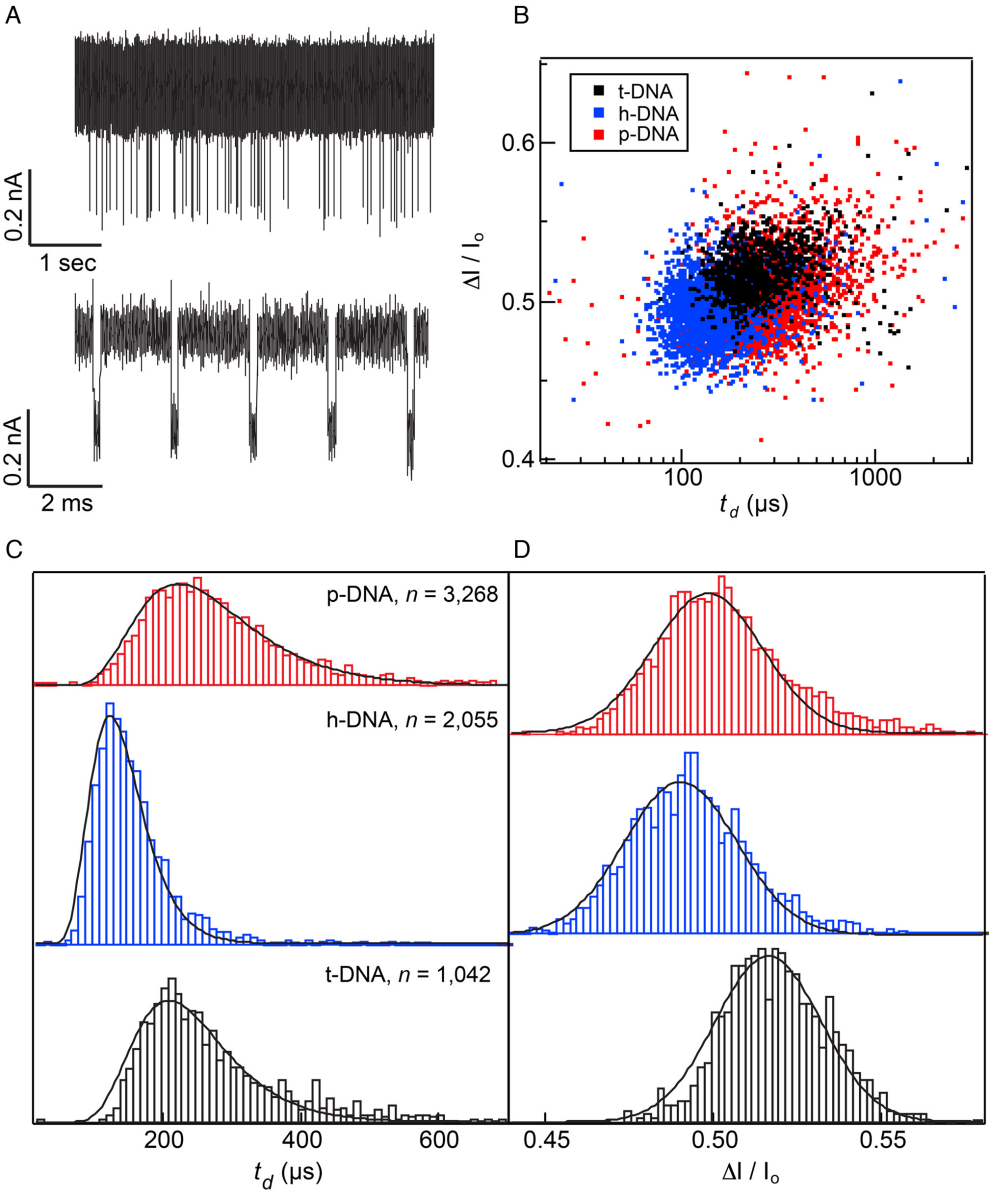
Translocation of thymine-modified DNA through a 2.8 nm diameter pore (effective pore thickness = 10 nm). (**A**) Raw current trace (*top*) and a concatenated set of events (*bottom*) obtained for t-DNA (traces filtered at 200 kHz). (**B**) Scatter plot of *ΔI/I_o_* versus *t_d_* for each DNA variant. (**C**) Normalized dwell time histograms for each DNA sample (event totals displayed). Histograms are fit to a 1D drift-diffusion model (see Table 1 for fit parameter details). (**D**) Fractional current blockade histograms of each DNA variant (Gaussian fits shown).

Figure 2 summarizes the results of DNA translocation experiments performed at *V* = 200 mV, 0.4 M KCl, pH 7.9 and *T* = 21°C (an additional data set for a smaller diameter pore is shown in Supplementary Figure S2). Figure 2A shows a continuous trace for t-DNA along with a sample of concatenated, consecutive events and Figure 2B displays the scatter plots that summarize our data. Dwell time histograms shown in Figure 2C reveal that h-DNA passes through the pore noticeably faster than both t-DNA and p-DNA, with the latter two molecules having similar mean dwell times. Fitting the dwell time distributions to a 1D drift-diffusion model (black curve) (19,44–46) yields two parameters, the drift velocity *v* and diffusion coefficient *D* (see Table 1). The presence of hmU modification increases the values of both *v* and *D*, while the mean current blockade decreases (Figure 2D). Considering the added oxygen within the hmU modification, we were surprised to find lower *ΔI/I_o_* values for that molecule, which implies that the larger steric footprint of an hmU nucleotide (which is expected to increase the current blockade) is overcompensated by some other effect that reduces the current blockade. Our observations are consistent with a model where a DNA containing hmU modifications has a more compact overall structure than its native T-form analog. Although steric exclusion is the most common mechanism of ionic current blockade, it is possible that changes in hydrophobicity and flexibility of DNA can account for the change in the ionic current blockade (10), as discussed below.

**Table 1.** Experimental and simulated translocation data for thymine-modified dsDNA.

Sample	*<t_d_>* (μs)	*v* (nm/μs)	*D* (nm^2^/μs)	(Δ*I / I_o_*)_E_	(Δ*I / I_o_*)_S_
*t*-DNA	293 ± 5	0.189 ± 0.003	0.45 ± 0.04	0.516 ± 0.022	0.477 ± 0.008
*h*-DNA	159 ± 2	0.325 ± 0.004	0.71 ± 0.07	0.490 ± 0.024	0.454 ± 0.007
*p*-DNA	309 ± 4	0.169 ± 0.002	0.53 ± 0.05	0.498 ± 0.023	0.471 ± 0.007

*Left*: Experimental mean dwell time *<t_d_>*, drift velocity *v*, diffusion coefficient *D* and fractional current blockade *ΔI/I_o_* determined by best fits of dwell time (1D drift-diffusion) and current blockade (Gaussian) histograms. *Right*: MD simulation results for *ΔI/I_o_*. For determination of uncertainties and details on the MD simulations, see Materials and Methods.

**Figure 3.**
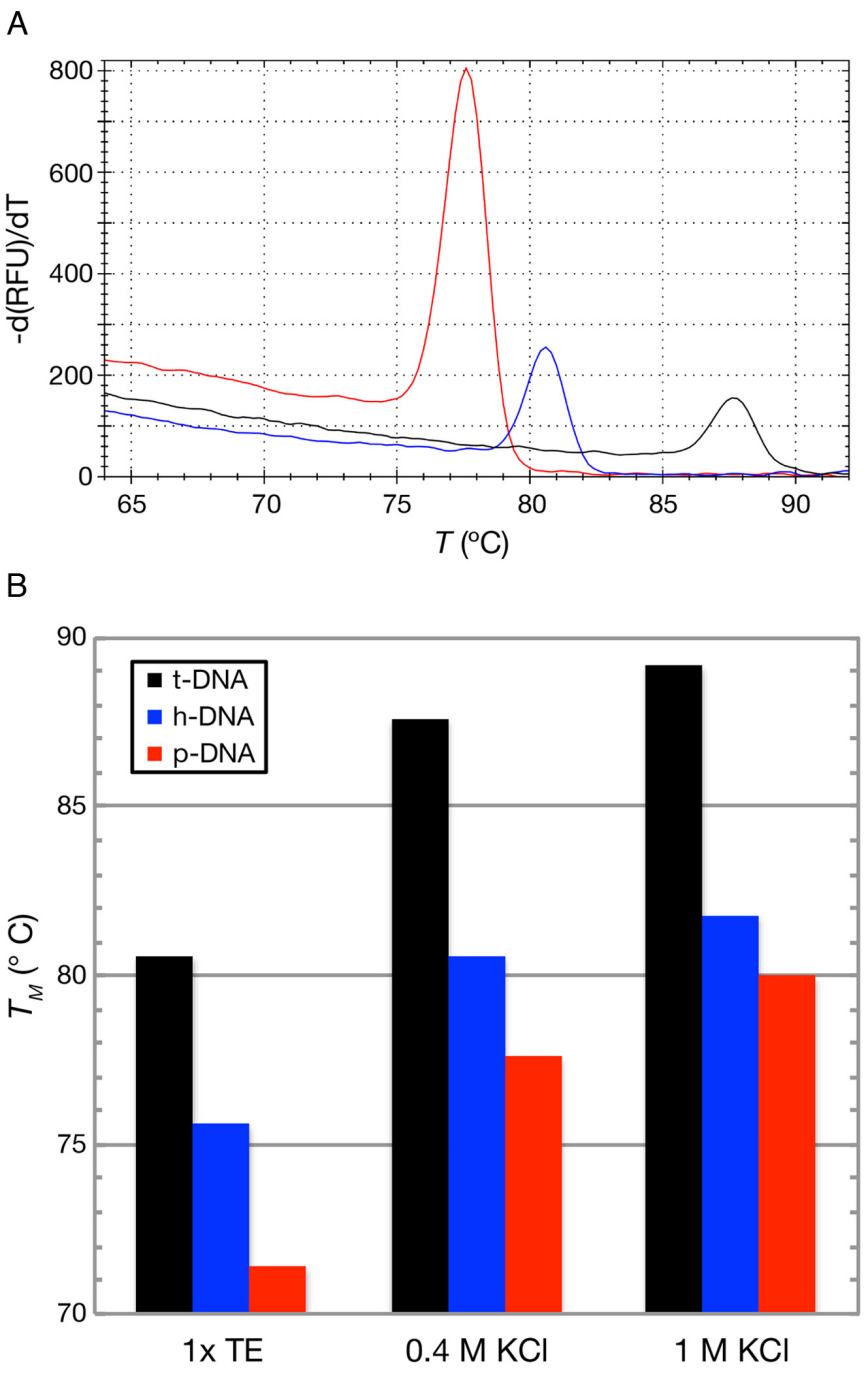
Melting curves of DNA variants for different buffer conditions. (**A**) Sample melting curves, measured as differential intercalator fluorescence versus temperature, for t-DNA (black), h-DNA (blue), and p-DNA (red) at the buffer conditions of 0.4 M KCl, 10 mM Tris, 1 mM EDTA at pH 7.9. (**B**) Melting curve peaks for buffer conditions shown.

To investigate the relative stability of our DNA variants, we determined the melting temperatures of each sample by collecting melting curves of the three DNA variants (Figure 3A; for protocol see www.northeastern.edu/wanunu/Protocols.php). From the peak of each differential fluorescence curve, we obtained the following melting temperatures for each sample in 0.4 M KCl buffer conditions: T_t-DNA_ = 87.6 ± 3.0°C, T_h-DNA_ = 80.6 ± 1.8°C, T_p-DNA_ = 77.6 ± 2.6°C. The melting curve data for the p-DNA sample are more sensitive to ionic strength than that of h-DNA, as shown in Figure 3B, which is consistent with the added charge of the modified base pair in that construct. The lower melting temperatures of h-DNA and p-DNA may be explained by the more hydrophilic structure of the grooves as compared with native t-DNA, which was found to be the case for the hmC modifications (10). However, the faster transport of h-DNA could not be explained directly using hydrophobic/hydrophilic considerations, as it is not clear what impact this parameter has on transport dynamics through a pore.

To elucidate the structural differences between the modified DNA strands, we performed all-atom MD simulations of 36-bp fragments of the 101-bp DNA constructs in 0.4 M KCl solution and in the absence of a solid-state nanopore (the nucleotide sequence of DNA used in the simulations is provided in Materials and Methods). Nine independent trajectories (three for each variant) of over 100 ns each were obtained in total. The trajectories were analyzed using the 3DNA program (47) that characterized the equilibrium conformation and structural fluctuations of the DNA constructs in terms of six local intra-base pair parameters (shear, buckle, stretch, propeller, stagger and opening) and six parameters defining the conformation of two neighboring base pairs (shift, tilt, slide, roll, rise and twist). Of these parameters, roll and twist describe bending of the double helix, whereas rise describes longitudinal stretching.

**Figure 4.**
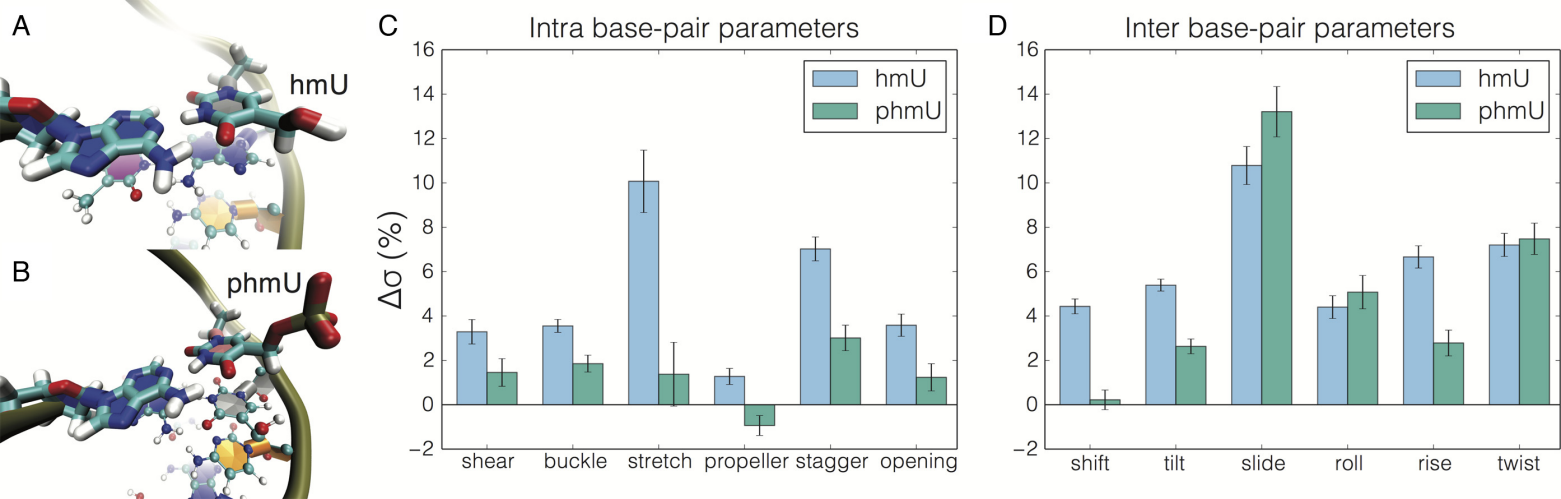
Results of molecular dynamics simulations characterizing structural fluctuations of the DNA variants. (**A**, **B**) Molecular bond representation of the hmU modification (A) and phmU modification (B) within the h-DNA and p-DNA constructs, respectively. (**C**) Differences in the standard deviation of the intra-base pair parameters for the hmU- and phmU-modified DNA relative to the values obtained for unmodified t-DNA. The h-DNA construct exhibits increased fluctuations in the average base pair geometry. (**D**) Differences in the standard deviation of the inter-base pair parameters for the h-DNA and p-DNA constructs relative to t-DNA. Both h-DNA and p-DNA show increased inter-base pair fluctuations suggesting that these molecules are more flexible than t-DNA. The relative difference in standard deviation Δσ_x_ = 100×(σ_x_ – σ_t_)/σ_t_, where σ_t_ and σ_x_ are the standard deviations of a parameter in t-DNA and x-DNA, respectively (x is either h or p), averaged over the time series and then over base pairs or base pair steps. The error was calculated by splitting MD trajectories of the same chemical system into thirty blocks and calculating the standard deviation within each block for each base pair or step. The standard error of these 30 estimates was then propagated to reflect averaging over all base pairs or steps.

We were primarily interested in the relative flexibility of the DNA constructs. Since the density of hmU modifications in the experimental constructs was very high (i.e. ≈60% of base pairs were modified), we predominantly focus here on structural fluctuations averaged over the entire constructs (Figure 4C,D), although the effect of point modifications was also addressed through simulations (Supplementary Figures S3 and S4). There are two modalities of flexibility in DNA: kinking and bending (48). During our ≈1 μs of simulation, we observed three kinking-like events (described in detail in the Supplementary Data), which did not allow us to draw a conclusion about the relative frequency of such events among the DNA variants. Thus, we restrict our analysis to the elucidation of the effect of hmU and phmU modifications on near-equilibrium structural fluctuations. Figure 4A,B shows typical near-equilibrium structures of the hmU and phmU modifications observed in our MD simulations.

The results of our analysis indicate that both hmU and phmU modifications have a pronounced effect on the standard deviations of the intra- and inter-base pair parameters, and hence on the elastic properties of the molecules (10). Figure 4C,D plots Δ*σ*, the relative difference in the standard deviation averaged over base pairs and trajectories, of each inter- and intra-base pair parameter for the two DNA variants with respect to the values observed for unmodified DNA (t-DNA). The h-DNA shows an increase in fluctuations for all of the intra-base pair parameters (Figure 4C), indicating increased flexibility of the DNA upon addition of hydroxyl groups. Similar changes in DNA flexibility were previously observed upon addition of hydroxyl groups to mC-modified DNA (10). The inter-base pair parameters also show a marked increase in fluctuations, as seen in Figure 4D, with all six parameters showing at least a 4% increase in the standard deviation over t-DNA. Specifically, the roll and twist parameters, which are closely linked to DNA bending, are 4.5% and 7.5% greater than in the t-DNA, indicating a greater bending ability of h-DNA. The standard deviation of the rise parameter is 7% greater in h-DNA than in t-DNA, indicating a greater propensity of h-DNA to stretch. Although large increases in the standard deviation of the intra-base pair parameters were not observed for p-DNA, the fluctuations of the inter-base pair parameters for p-DNA were similar to those of h-DNA, suggesting similar flexibility of the two constructs. The latter result is perhaps not surprising, as our p-DNA molecule contained a large number of hmU modifications in addition to phmU modifications. Similar increases of the standard deviations were observed in our MD simulations of DNA constructs containing individual hmU modifications (Supplementary Figure S3). In contrast to hmU, individual phmU modifications reduced the standard deviation of the structural parameters, making the DNA more rigid (Supplementary Figure S4).

**Figure 5.**
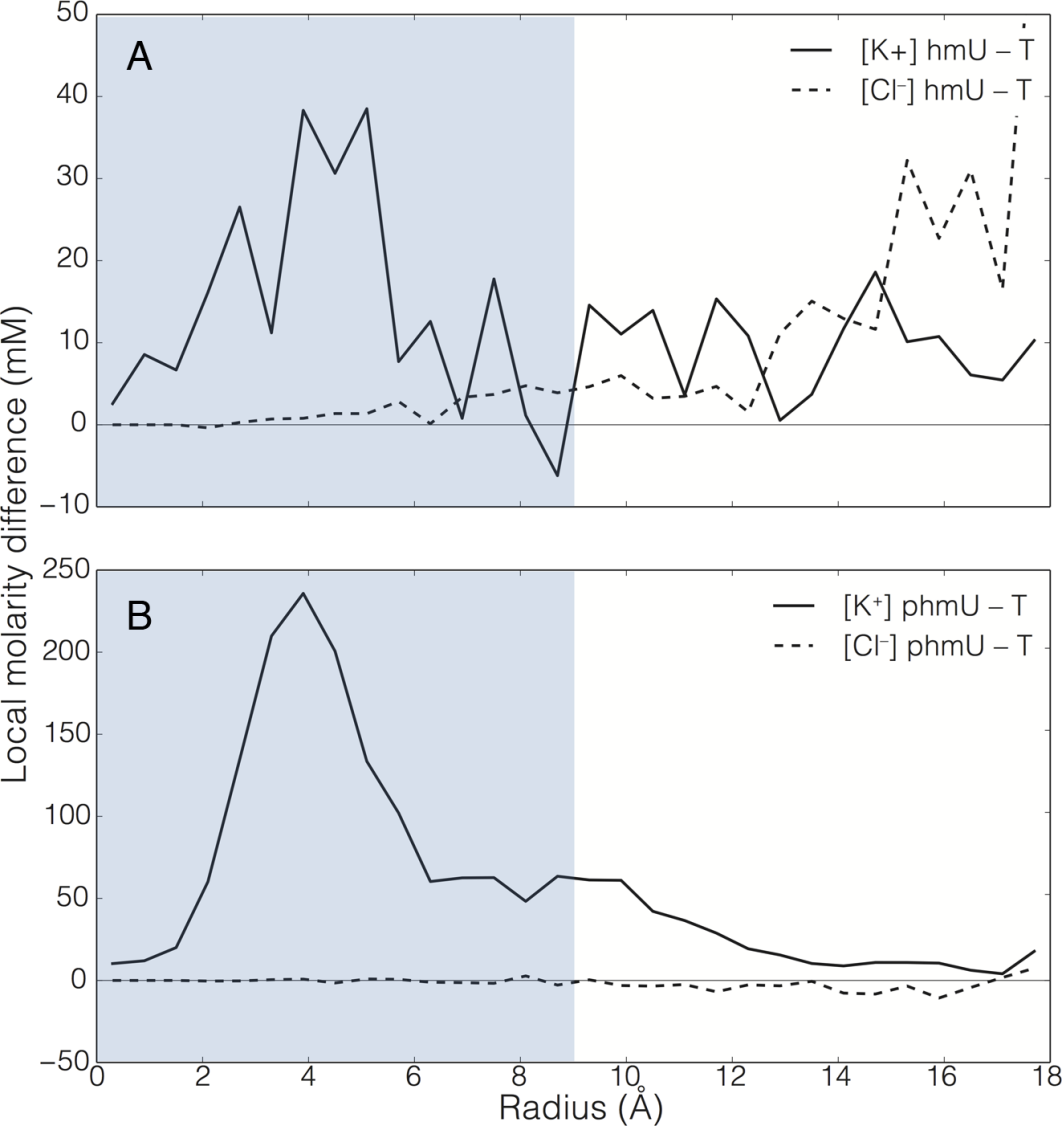
MD simulations of ion atmosphere in a solid-state nanopore occupied by DNA variants. (**A**) The effect of hmU modification on the distribution of K^+^ (solid line) and Cl^−^ (dashed line) ions in a 3.4 nm diameter nanopore. The local concentration difference between the h-DNA and t-DNA systems (black lines) is plotted as a function of the radial distance from the pore axis. The horizontal line passing through zero is a guide to eyes. (**B**) Same as in panel (A), but for the p-DNA and t-DNA systems. The regions occupied by DNA are indicated by blue rectangles.

MD simulations of the DNA constructs in a solid-state nanopore provided further insights into our experimental observations. For these simulations, we constructed four atomic-scale models of the experimental system, each containing a 3.5 nm diameter nanopore in a 7 nm-thick SiN membrane, as shown in Figure 1A (see Materials and Methods for simulation details). One of the models contained a nanopore filled with 0.4 M KCl (open pore system), while the other three also contained a 36-bp fragment of the 101-bp construct used in experiment inserted into the pore. Each system was subject to an external electric field producing a 200 mV bias across the membrane resulting in an ionic current that was measured (41) (see Supplementary Data for details). For each variant of DNA, we ran five independent simulations of 120 ns each. To minimize the effect of conformational noise, the DNA constructs were restrained to adopt the same conformation in the nanopore, allowing us to obtain an accurate estimate of the ionic currents. The ionic current data were first averaged in 20 ns blocks, and then the mean current and standard error were computed by averaging over 30 such blocks. The results of the applied field simulations indicated a 4.5% increase in the current when all T were replaced by hmU, which was accompanied by a 5.2% average increase in the number of mobile ions (not bound to the pore walls) within the pore constriction (Figure 5A). When comparing h-DNA with p-DNA, the number of potassium ions is higher in the pore for p-DNA (Figure 5B). This increase, however, is fully explained by the added charge of the phosphorylated base (–2e^−^), while no change in chloride density in the pore was observed. While simulated fractional current blockades *ΔI/I_o_* qualitatively agree with the experimental trend h-DNA < p-DNA < t-DNA (see Table 1), it is the combination of the DNA structural dynamics and the ion distribution in the nanopore that accounts for the experimental translocation data.

In conclusion, our combined results suggest that the presence of a hydrophilic hmU modification in place of T enhances DNA flexibility. Thus, our nanopore measurements indicate faster translocation of h-DNA in comparison to t-DNA, while MD simulations indicate that h-DNA is more flexible both laterally and longitudinally than t-DNA. This enhanced flexibility of h-DNA renders its navigation through the steric constraints of a narrow pore more efficient than that of a stiffer molecule, which explains the faster translocation. This situation is in contrast to the behavior expected for large pores and long DNA constructs, where increased flexibility of a molecule is expected to make its translocation through a nanopore slower due to increased polymer entropy (45). Enhanced longitudinal flexibility of h-DNA and p-DNA could have an additional effect on the ionic current blockades by reducing the effective diameter of the molecules when they are subject to the stretching force of the electric field in a nanopore. Overall, our study suggests that oxidation of DNA bases can affect DNA mechanical properties and that such modifications can be studied by nanopore translocation measurements. These findings suggest a role of oxidized DNA bases on the deformability and ionic environment of the grooves of modified DNA, which may modulate the binding of regulatory and repair proteins.

## Supplementary Material

SUPPLEMENTARY DATA
