# Supplementary material for: Hydroxymethyluracil modifications enhance the flexibility and hydrophilicity of double-stranded DNA
Source: Nucleic Acids Res. 2015 Nov 17;44(5):2085–92. doi: 10.1093/nar/gkv1199 (PMC4797264; doi:10.1093/nar/gkv1199)
Supplement: SUPPLEMENTARY DATA [file supp_44_5_2085__index.html]

Hydroxymethyluracil modifications enhance the flexibility and hydrophilicity of double-stranded DNA — SUPPLEMENTARY DATA 

# Hydroxymethyluracil modifications enhance the flexibility and hydrophilicity of double-stranded DNA

## SUPPLEMENTARY DATA

- SUPPLEMENTARY DATA
- SUPPLEMENTARY DATA
- SUPPLEMENTARY DATA
