# Supplementary material for: Hydroxymethyluracil modifications enhance the flexibility and hydrophilicity of double-stranded DNA
Source: Nucleic Acids Res. 2015 Nov 17;44(5):2085–92. doi: 10.1093/nar/gkv1199 (PMC4797264; doi:10.1093/nar/gkv1199)
Supplement: SUPPLEMENTARY DATA [file supp_gkv1199_nar-01941-f-2015-File007.doc]

**Supplementary Data for**

**Hydroxymethyluracil Modifications Enhance the Flexibility and Hydrophilicity of Double-Stranded DNA**

Spencer Carson^1^, James Wilson^2^, Aleksei Aksimentiev^2^, Peter R. Weigele*^,3^, and Meni Wanunu*^,1,4^

^1^Department of Physics, Northeastern University, Boston, Massachusetts, USA

^2^Department of Physics, University of Illinois at Urbana-Champaign, Urbana, Illinois, USA

^3^New England Biolabs, Ipswich, Massachusetts, USA

^4^Department of Chemistry/Chemical Biology, Northeastern University, Boston, Massachusetts, USA

* To whom correspondence should be addressed. Tel: +1 617 373 7412; Fax: +1 617 373 2943; Email: [wanunu@neu.edu](mailto:wanunu@neu.edu)

Correspondence may also be addressed to Tel: +1 978 380 7304; Fax: +1 978 921 1350; Email: [weigele@neb.com](mailto:weigele@neb.com)

**Table of Contents:**

Figure S1. Sequence and restriction map of amplicon used in this study………………………………...2

Figure S2. Nanopore translocation dataset for thymine-modified DNA samples in a smaller pore........3

Figure S3. The effect of point hmU modifications on DNA flexibility……………………………………….4

Figure S4. The effect of point phmU modifications on DNA flexibility …………...……...………………...5

SD-1. Abnormal DNA conformations encountered during MD simulations….………..……………...…...6

SD-2. Simulations of DNA variants containing point hmU modifications …………….…………………...6

SD-3. Determination of local flexibility of DNA for individual phmU modifications ……………….……...6

SD-4. Determination of ionic current and molarity from MD simulations…………….……………………7

SD-5. References……………………………………………………………………………………………….7

Figure S1. Sequence and restriction map of the amplicon used in this study.


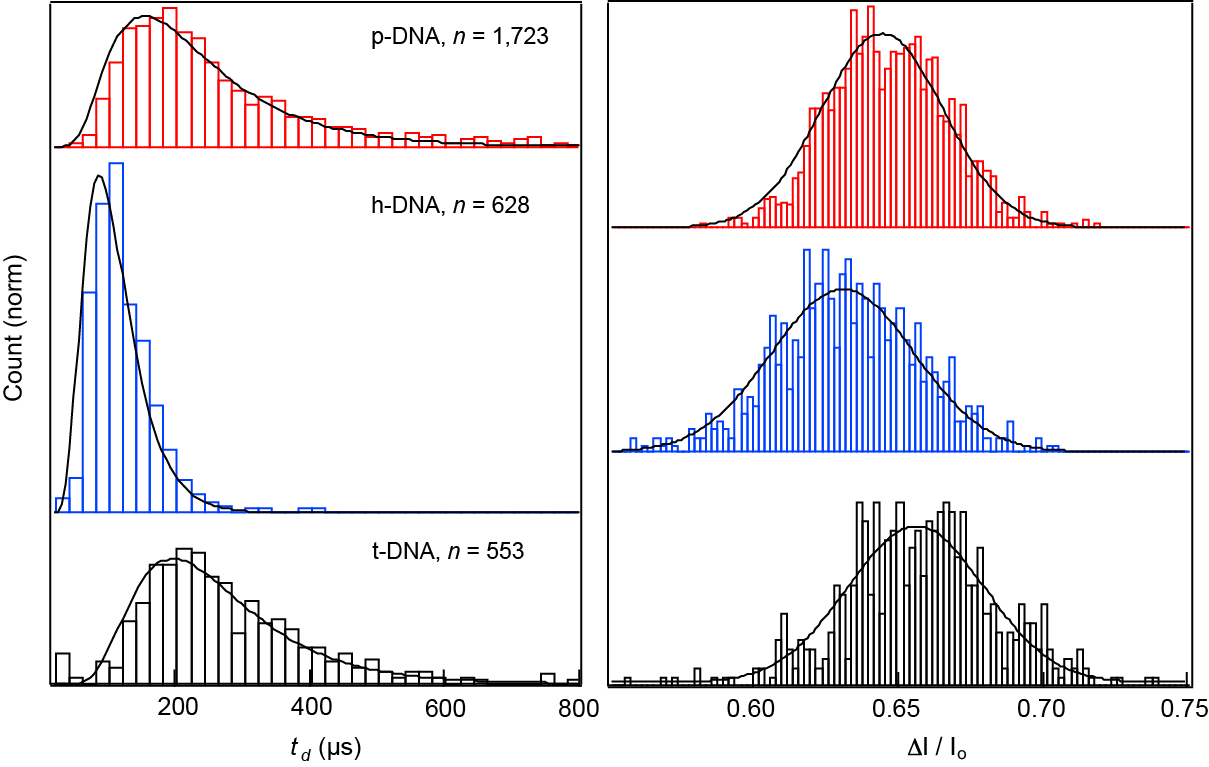


Figure S2. Nanopore translocation dataset for thymine-modified DNA samples obtained using a nanopore smaller in diameter than the one reported in the main text (Fig. 2). Dwell time and fractional current blockade histograms for modified DNA measured using a 2.6 nm diameter nanopore (*V* = 200 mV, 0.4 M KCl, pH 7.9, *T* = 21°C). All trends observed in this set of measurements correlate with the data reported in the main text. The order of data collection was t-DNA, h-DNA, p-DNA, which differs from the dataset shown in Fig. 2 (t-DNA, p-DNA, h-DNA).

Figure S3. The effect of point hmU modifications on simulated DNA. Differences in the standard deviation of the intra-base pair (*left*) and inter-base pair (*right*) parameters for the hmU-modified DNA relative to the values obtained for unmodified t-DNA. In contrast to the simulations reported in the main text Figure 4, here we analyze free-equilibration trajectories of DNA constructs containing point hmU mutations (see SD-2 note below). The relative difference in standard deviation Δσ_h_ = 100×(σ_h_ – σ_t_)/σ_t_, where σ_h_ and σ_t_ are the standard deviations of a parameter in h-DNA and t-DNA, respectively, averaged over the time series, and then over base pairs or base pair steps containing the point hmU modifications. The error was calculated by splitting MD trajectories of the same chemical system into thirty blocks and calculating the standard deviation within each block for each base pair or step. The standard error of these 30 estimates was then propagated to reflect averaging over all base pairs or steps containing point hmU modifications.

Figure S4. The effect of point phmU modifications on simulated DNA flexibility. Differences in the standard deviation of the intra-base pair (*left*) and inter-base pair *(right)* parameters for the phmU-modified DNA relative to the values obtained for h-DNA. The relative difference in standard deviation Δσ_p_ = 100×(σ_p_ – σ_h_)/σ_h_, where σ_p_ and σ_h_ are the standard deviations of a parameter in p-DNA and h-DNA, respectively, averaged over the time series, and then over base pairs or base pair steps containing the phmU modifications. The error was calculated by splitting MD trajectories of the same chemical system into thirty blocks and calculating the standard deviation within each block for each base pair or step. The standard error of these 30 estimates was then propagated to reflect averaging over all base pairs or steps containing phmU modifications.

**SD-1. Abnormal conformations of dsDNA encountered during MD simulations**

During the free equilibration simulations of DNA variants, DNA was seen to assume abnormal conformations in three separate instances. Two of these abnormal conformations occurred during a single simulation of p-DNA, and another occurred during a simulation of t-DNA.

In one of the three simulations of the t-DNA system, the DNA backbone assumed a sharp, 90-degree bend at base 8 (T·A) and 9 (C·G) after ~ 90 ns of equilibration. The DNA maintained this abnormal conformation for the remaining 30 ns of the equilibration and the base pairing pattern was not broken. The presence of the kink considerably increased the twist (steps 8,9), propeller (base 8), and slide (steps 7,8,9) parameters of the base pairs involved. As this abnormal conformation was not observed in the previous microsecond simulations of canonical DNA, we assumed it to be a rare event and excluded this part of the equilibration trajectory from our analysis.

During the free equilibration simulation of p-DNA, two abnormal conformations occurred at two different sites on the molecule. Base pair 25 (A·hmU base pair) was no longer hydrogen bonded after *t* = 89 ns, which resulted in a staggered stacking of the bases. The rupture widened so that base 26 (phmU·A base pair) was no longer hydrogen bonded after *t* = 104 ns. Another DNA base pair ruptured in the same p-DNA simulation at *t* = 85 ns at base 14 (A·hmU base pair), but the hydrogen bonds reformed by *t* = 95 ns. At the kinks, DNA was no longer in a near-equilibrium configuration. Furthermore, the base pair pattern was broken, which is incompatible with the geometry assumed by the DNA conformation analysis algorithm. Hence, we excluded from our analysis the last 35 ns of the free equilibration trajectory of the p-DNA system where the two kinking events were observed.

**SD-2. Simulations of DNA variants containing point hmU modifications**

To measure the structural effect of point modifications of T into hmU, we created four copies of the t-DNA system, and in each we modified one T (at position 22, 24, 25 and 26) into hmU. We then simulated these singly modified constructs in free solution for 120 ns each. Following that, we measured the structural parameters for only the modified base pairs or steps (Fig. S3). The increased fluctuations of both intra- and inter-base pair parameters (with the exception of shear which shows no change) suggests a greater local flexibility of an hmU-containing DNA fragment in comparison to unmodified DNA.

**SD-3. Determination of local flexibility of DNA for individual phmU modifications**

Since the phmU modifications were not as dense as hmU modifications in the constructs used for our free equilibrium simulations, we were able to use our original free equilibration trajectories to determine the structural effects of point modification of hmU into phmU. From the simulations of p-DNA in free solution, we measured the structural parameters of the modified bases, and compared them to the corresponding parameters measured in the h-DNA construct. The results of this analysis (Fig. S4) indicate smaller fluctuations of all intra-base pair parameters in the phmU-containing DNA fragments in comparison to the hmU-containing fragments. The effect of phmU modification on the fluctuations of the inter-base pair parameters was mixed: slide, roll, and twist increased whereas shift, tilt, and rise decreased. We interpret these results as an indication of an overall decreased flexibility of phmU-containing DNA in comparison to DNA carrying only hmU modifications.

**SD-4. Determination of ionic current and molarity from MD simulations**

To compute the ionic current blockade, the open nanopore system (which had no DNA in it) was first simulated for 45 ns at a 200 mV transmembrane voltage. Following that, five independent simulations of 60 ns were performed using different random seeds to measure the open pore current.

For all nanopore systems, the instantaneous ionic current was computed as

$$I(t)=\frac{1}{\Delta tL_{z}}\sum_{i} q_{i}[z_{i}(t+\Delta t)-z_{i}(t)],$$

where *q_i_* and *z_i_* were the charge and z-coordinate of ion *i*, respectively, and *Δt* was 10 ps. The current was measured in a 2 nm thick slab (*i.e.*, *L_z_* = 2 nm) centered on the midplane of the pore. The average current was calculated by taking the mean of the current series. To calculate the error in the current values, the current data was split into 20 ns blocks from which a mean value was computed, and then the mean and standard error from these blocks were calculated.

Molarity was calculated by counting the average number of K^+^ or Cl^–^ ions in a 3 nm tall cylindrical shell centered on the pore axis and then dividing by the volume of the shell. The ion molarity difference between the h- or p-DNA and t-DNA systems, Fig. 5, was computed by subtracting the local ion molarity in the unmodified DNA system from the local ion molarity in the modified DNA system.

**SD-5. References**

1. Galindo-Murillo, R., Roe, D.R. and Cheatham III, T.E. (2014) On the absence of intrahelical DNA dynamics on the μs to ms timescale. *Nature communications*, **5**.5152
